# Hericium ophelieae sp. nov., a novel species of Hericium (Basidiomycota: Russulales, Hericiaceae) from the Southern Afrotemperate forests of South Africa

**DOI:** 10.1080/21501203.2023.2191636

**Published:** 2023-03-21

**Authors:** B. Van der Merwe, P. Herrmann, Karin Jacobs

**Affiliations:** a Department of Microbiology, Stellenbosch University, Private Bag X1, Stellenbosch, South Africa; b Harmonic Mycology, 6 Nthombeni Way, Noordhoek, South Africa

**Keywords:** Russulales, *Hericiaceae*, taxonomy, novel species, ITS

## Abstract

A novel species of *Hericium* was recently collected in the Afrotemperate forests (Knysna – Amatole region) of Southern Africa. The novel species shares many similar, dentate features common to other species in *Hericium*, and its basidiome first appears stark white and yellows with age. However, the substrate choice and gloeocystidia and basidiospore sizes of the specimens collected were distinct from other *Hericium* species. This was confirmed by sequencing the ITS and 28S genetic markers, respectively. The novel species is described as *Hericium ophelieae* sp. nov. and appears unique as it grows on hardwoods indigenous to Southern Africa. The species has larger basidiospores and wider gloeocystidia compared to its closest relative. *H. ophelieae* sp. nov. is the first endemic species of the medicinal mushroom genus *Hericium* to be described from Southern Africa, and the second to be described from Africa, after its closest relative, *H. bembedjaense*, which was isolated in Cameroon. Although this is the first *Hericium* to be described from the Southern African region, there are likely others to be discovered, and this study highlights the need for further research into the fungal diversity of Afrotemperate environments.

## Introduction

The family *Hericiaceae* (*Russulales, Basidiomycota*) consists of the three genera *Hericium* (Scop.) Pers, *Laxitextum* (Pers.) Lentz, and *Dentipellis* Donk (Das et al. 2011). The genus *Hericium* is currently represented by 28 species (Jumbam et al. 2019) and was first described by Schrank in 1786 as *Hericiaceae* (Index Fungorum, 2023). It was renamed by Persoon, who in 1794 named *Hericium coralloides* (Scop.) Pers as the type species for the genus (Persoon 1794). Despite the comparative rarity of the genus in nature, the number of known species and the known distribution of the genus has expanded significantly, with the most recent species described by Jumbam et al. (2019) and Singh and Das (2019). To date, two species of *Hericium* have been recorded in Africa, namely *H. erinaceus* (Bull.) Persoon (Ouali et al., 2020) and *H. bembedjaense* Jumbam (Jumbam et al. 2019), although *H. erinaceus* is found on other continents such as Asia and North America. *H. bembedjaense* was described from a specimen found in the broadleaf tropical forests of the Dja Biosphere reserve, Cameroon. *H. bembedjaense* is the only endemic African species of this genus described to date, although there are very likely other species that are yet to be discovered (Jumbam et al. 2019). However, progress has been hindered by the lack of studies on African *Russulales* (Jumbam et al., 2019) and fungi in general, while the cryptic morphology of *Hericium* species makes it difficult to distinguish between species (Hallenberg, Nilsson, and Robledo, 2013). Molecular analysis techniques are now more regularly used in the identification and description of new species.

Members of the genus *Hericium* are a relatively rare sight in nature (Singh and Das 2019). These coral-like, dentate basidiomycetes are white rot decomposers. Ecologically, *Hericium* species play an important role in the breakdown of hardwoods and conifers (Jumbam et al. 2019) and can be found growing on both standing or fallen dead trees (Boddy et al. 2011).

*Hericium* species are often cultivated for their medicinal and culinary values. Historically, *H. erinaceus* has long been part of traditional Chinese and Asian medicine (Ghosh et al. 2021). The metabolites produced by both the fruiting bodies and mycelia of some members of the genus *Hericium* are of particular interest due to their bioactive nature. Several of the compounds produced – such as hericenones, erinacines, and corallocins -, have the potential to treat a variety of ailments with negligible side effects (Ma et al. 2010; Wittstein et al. 2016; Jumbam et al. 2019). For example, *H. erinaceus* has been shown to treat neuropsychiatric disorders like depression (Chong, Fung, Wong and Lim, 2020), while *H. novae-zealandiae* has anticancer properties (Chen et al. 2019). *H. coralloides* was found to have anti-ageing and antioxidant properties (Zhang et al., 2019).

Recently, specimens believed to be *H. coralloides* were collected from hardwoods indigenous to the Knysna – Amatole Southern Afrotemperate region of South Africa (Mucina and Rutherford, 2006). However, based on phylogenetic analysis and differences in morphology, it is believed that these specimens represent a previously undescribed species. In this paper, this novel species was characterised based on phylogenetic analysis and morphological characteristics and described as *Hericium ophelieae* sp. nov.

## Materials and Methods

### Morphological study

Fruiting bodies were sporadically collected for three years (2019–2021) between December and March from the Knysna – Amatole Southern Afrotemperate region. The specimens for this species were collected from *Rapanea melanophloeos* (L.) Mez, *Ilex mitis* (L.) Radlk., *Olea capensis* Lam., and *Ocotea bullata* (Burch.) E. Meyer host trees. The macro features of the fresh basidiomata were noted. Fresh specimens were photographed in the field, collected, and then preserved for further study by freezing at −20°C or by drying using silica gel.

For the micromorphological studies, dried fruiting bodies were hydrated using 3% KOH. Tissue samples were stained using Congo Red (Pallua et al. 2012) and fixed to slides using Shears mounting fluid. The basidiospores, gloeocystidia, and basidia were observed, measured, and drawn under a differential interference contrast microscope (Nikon Eclipse E800, Japan) with a CFI plain Apochromat VC 100X lens. At least 20 examples for each feature were examined and measured at 400X and 1000X magnification. Spore sizes were measured in length (L) and width (W) with the spores in side profile. Calculations were made as (L_a –_ L_b_ – L_c_) and (W_a_ – W_b –_ W_c_) where X_a_ = smallest measurement, X_b_ = average of the measurements and X_c_ = the largest measurement (Jumbam et al. 2019).

### DNA extraction, PCR amplification, and sequencing

Genomic DNA from both dried *Hericium* basidiomata and cultured mycelia was extracted using ZR Quick-DNA Fungal/Bacterial Miniprep Kits (Zymo Research, USA). Approximately 100 mg of fungal tissue was used per extraction and extractions were carried out according to the manufacturer’s instructions. Successful DNA extractions were visualised on a 1% agarose gel with ethidium bromide. DNA was then kept at −20°C for storage until Polymerase Chain Reaction (PCR) reactions were performed.

A PCR amplification was performed on the extracted DNA, using universal primers to target the Internal Transcribed Spacer (ITS) and 28S ribosomal RNA regions. For ITS, ITS1 (5’-TCCGTAGGTGAACCTGCG-3’) and ITS4 (5’-TCCTCCGCTTATTGATATGC-3’) primers were used, and LR0R (5’-ACCCGCTGAACTTAAGC-3’) and LR6 (5’-CGCCAGTTCTGCTTACC-3’) were used for the 28S region (Moncalvo, François M. Lutzo, 2000; Vilgalys and Hester 1990; White et al. 1990; Jumbam et al. 2019). Each 10 μl amplification reaction consisted of 4.1 μl MilliQ water, 5 μl KAPA *Taq* ReadyMix 2X (Sigma-Aldrich, USA), 0.2 μl forward and reverse primers (0.2 mM), and 0.5 μl of 20 mg/μl extracted DNA.

The PCR runs were carried out on a 2720 Thermal Cycler (Thermo Fisher Scientific, USA) using the following parameters. For the ITS region, the initial denaturing at 94°C for 5 minutes, followed by 30 cycles at 94°C for 30 seconds, 56°C for 30 seconds, and a final extension at 72°C for 7 minutes. The samples were held at 4°C. For 28S, the initial denaturing happens at 94°C for 5 minutes, followed by 40 cycles at 94°C for 45 seconds, 54°C for 45 seconds, and a final extension at 72°C for 6 minutes. The samples were also held at 4°C.

Successful PCRs were visualised using a 1% agarose gel, with ethidium bromide. A sequence PCR reaction was set up with the successful PCRs. Each 10 μl sequence reaction consists of 1 μl amplified DNA, 1.25 μl Buffer, 1 μl BigDye, 1 μl forward primer (0.2 mM) (ITS1 or LR0R, respectively), and 5.75 μl MilliQ water. The sequence reaction was performed on the 2720 Thermal Cycler, with initial denaturing at 96°C for 1 minute, followed by 25 cycles at 96°C for 10 seconds. The annealing temperature was set at 50°C for 10 seconds and extension step was 60°C for 4 minutes. The sequence reaction products were subsequently analysed at the Central Analytical Facility (CAF) using an ABI3730xl sequencer (CAF, Stellenbosch University).

### Phylogenetic analysis

The sequences obtained were trimmed using Chromas 2.6.6 (Technelysium, DNA Sequencing Software, Australia) for quality control, after which they were deposited at the National Center for Biotechnology Information’s (NCBI) database, GenBank. The accession numbers OP458323, OP458324, and OP458325 were assigned to the three sequences representing the ITS data, and OP458326, OP458327, and OP458328 were assigned to the 3 sequences representing the 28S data. Subsequently, the sequences were compared to similar species on the NCBI GenBank database using the nucleotide Nucleotide BLAST algorithm of 2022 (Altschul et al., 1997). ITS and 28S regions of similar *Hericium* species as well as *Dentipellis leptodon*, the designated outgroup, were downloaded. A multiple alignment was performed on the 21 ITS sequences (Table 1) using Geneious Alignment (Geneious Prime 2021.2.2). The aligned sequences were then trimmed, and ambiguous regions were removed prior to tree construction. The software package PAUP v. 4.0a (Swofford 2002) was used to construct the phylogenetic trees. Phylogenetic analysis was performed using a maximum parsimony with a heuristic search. Characters were treated as unweighted with gaps treated as missing data. Maxtrees were set at 500 trees. Branch swapping was done through stepwise addition, using a TBR algorithm, swapping on best trees only. Tree length (TL), consistency index (CI), retention index (RI), rescaled consistency index (RC), and Homoplasy index (HI) were calculated. Confidence was calculated using bootstrap analysis of 1 000 replicates using a full heuristic search. The analysis was run with the model TPM2uf+G selected by AICc with jModeltest 0.1.1 (Guindon and Gascuel 2003; Darriba et al. 2012). All trees were saved in Treebase (30179). A Bayesian analysis was run using MrBayes v. 3.2.634 (Ronquist et al. 2012). The analysis was run with the model K80 + G selected by BIC with jModeltest 0.1.1 (Guindon and Gascuel 2003; Darriba et al. 2012). The analysis included four parallel runs of 5000000 generations, with a sampling frequency of 1000 generations. The posterior probability values were calculated after the initial 25% of trees were discarded as the chains converged after 118600 generations.

**Table 1.** List of ITS sequence accession numbers from GenBank used in the phylogenetic analysis, country of origin is specified as far as known. Type sequences marked with an asterisk.

Species	Country	ITS	Authors/references
*Dentipellis leptodon*	New Zealand	MN044064.1	Cooper, J.A. and Smith, C.A. Direct Submission
*Hericium abietis*	Canada	AY534579.1	Park et al. 2004
*Hericium abietis*	N/A	JN201334.1	Eberhardt, U. Direct Submission
*Hericium alpestre*	Germany	MK491173.1	Buettner, E. and Kellner, H. Direct Submission
*Hericium alpestre*	N/A	MW787010.1	Bisko et al. Direct Submission
*Hericium bembedjaense*	Cameroon	MK683483.1	Jumbam et al. 2019
Hericium bembedjaense*	Cameroon	NR173834.1	Jumbam et al. 2019
*Hericium coralloides*	Russia	MG735348.1	Senik, S.V. et al. Direct Submission
*Hericium coralloides*	USA	MT759716.1	Rodgers, T. Direct Submission
*Hericium erinaceus*	N/A	MW131236.1	Qi, J et al. Direct Submission
*Hericium erinaceus*	N/A	MW131234.1	Qi, J et al. Direct Submission
*Hericium_novae-zealandiae*	New Zealand	MW862789.1	Weir, B.S. and Park, D. Direct Submission
*Hericium_novae-zealandiae*	New Zealand	MN044068.1	Weir, B.S. and Park, D. Direct Submission
*Hericium ophelieae*	South Africa	OP458323	This paper
*Hericium ophelieae*	South Africa	OP458324	This paper
Hericium ophelieae*	South Africa	OP458325	This paper
*Hericium rajendrae*	India	MH890521.1	Singh and Das 2019
*Hericium rajendrae*	India	MH890520.1	Singh and Das 2019
Hericium rajendrae*	India	NR169943.1	Singh and Das 2019
*Hericium yumthangense*	China	MH085972.1	Wang, M. Direct Submission
*Hericium yumthangense*	China	MH085971.1	Wang, M. Direct Submission

## Results

Blast results of the ITS and 28S sequences confirmed that the fruiting bodies collected in the Knysna – Amatole Afrotemperate forest matched those of the genus *Hericium*. The ITS sequences showed highest similarity to *H. bembedjaense*, with a percentage identity of 98.86% (Jumbam et al. 2019). The LSU sequences were not used for phylogenetic analysis as the availability of reference sequences for this gene in the genus *Hericium* is very limited, and this region did not resolve a number of the known species of *Hericium* and were not used for further analysis. It was, however, interesting to note that the species from Africa clustered separately from other species in *Hericium*. The dataset for the ITS analysis contained 668 characters of which 535 characters were constant, 59 variable characters were parsimony-uninformative and 74 characters parsimony-informative characters. A total of 52 trees were retained, with tree length  = 168, CI = 0.893, RI = 0.938, RC = 0.838, and HI = 0.107. The tree generated from the analysis of the ITS dataset showed that the African strains formed a separate clade, and *H. ophelieae* sp.nov. and *H. bembedjaense* grouped in separate clades with high bootstrap and posterior probability values.

### Taxonomy

**Figure 1.**
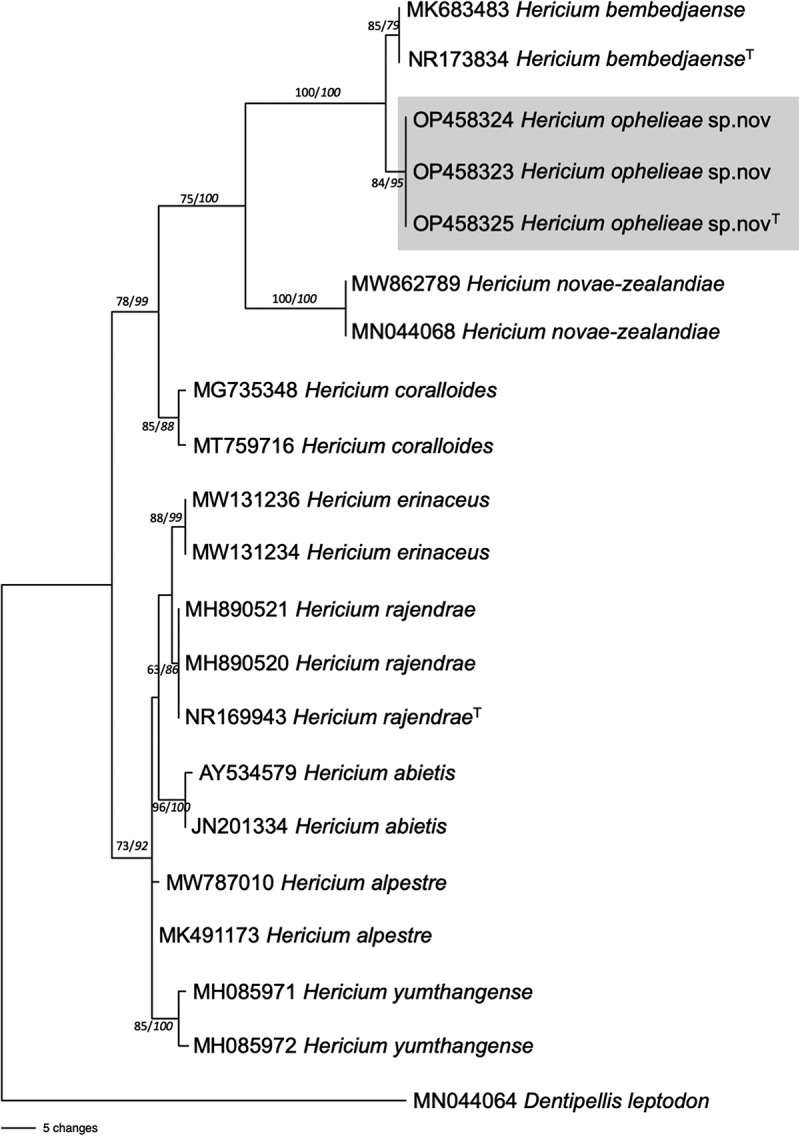
Phylogenetic tree inferred from the ITS dataset. Sequences are referred to by their species name and GenBank accession number. Bootstrap values are indicated as the first value on the branch followed by the probability value in italics. ^T^ indicates a type strain.

**Figure 2.**
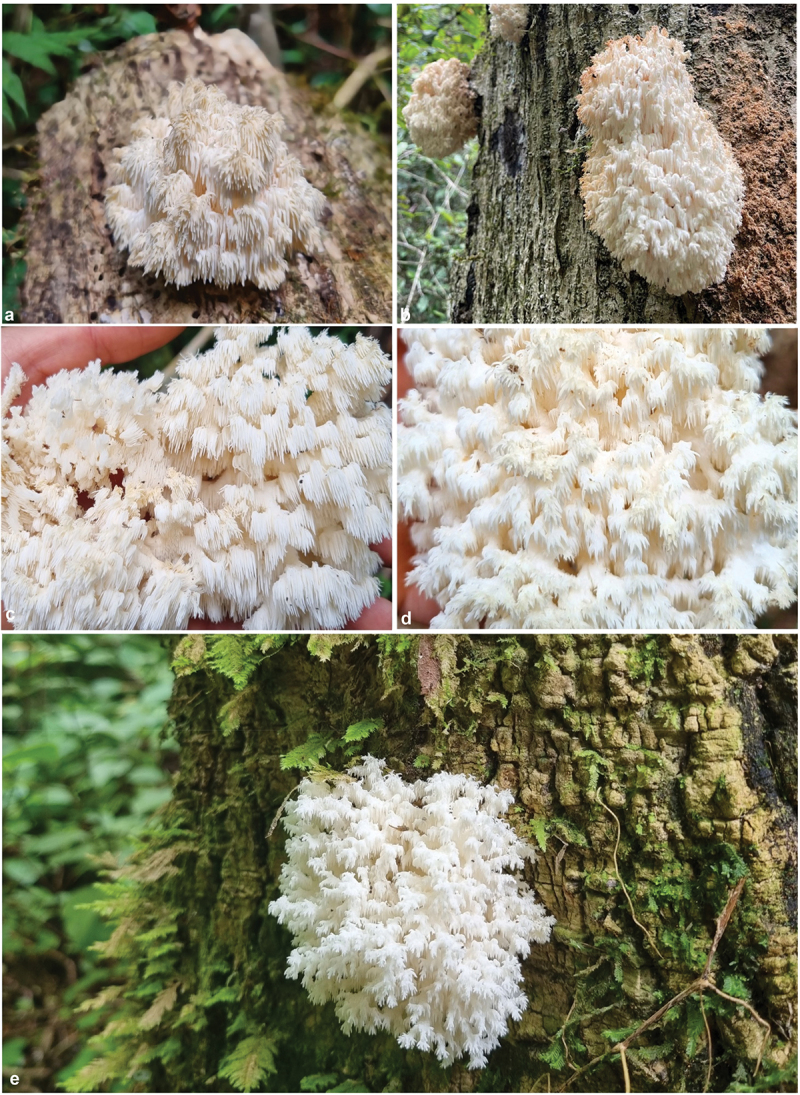
Plates **A**–**E** showing the macro features of *Hericium ophelieae* basidiomes in the field (10 cm to 30 cm in width), at varying stages of development (Photographs: P. Herrmann).

**Figure 3.**
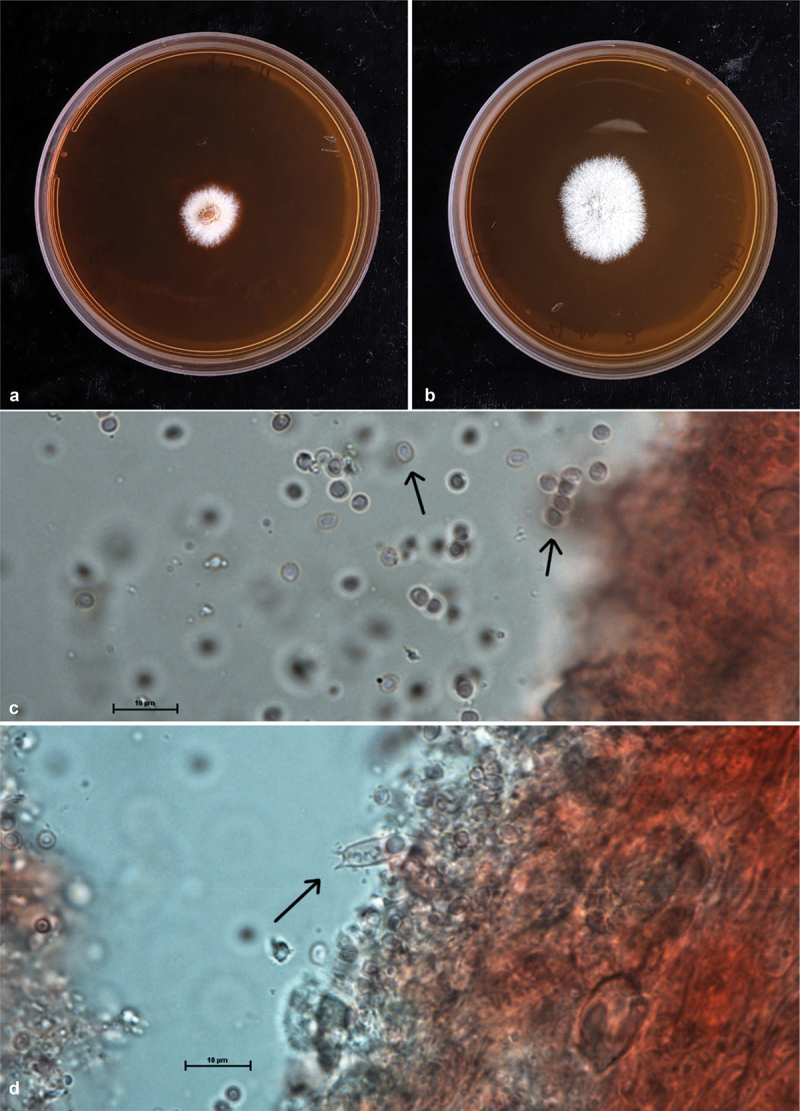
Plates **a, b** showing two strains of *Hericium ophelieae* growing on 90 mm malt extract agar Petri Dishes after 14 days of growth at 26°C. Plate **c** basidiospores at 100X magnification, enhanced with Congo Red. Plate **d** basidia, at 100X magnification, enhanced with Congo Red. Scale bars = 10 μm. (Photographs: T. Conradie, B. Van der Merwe).

*Hericium ophelieae* Van der Merwe and Jacobs sp. nov. *–* (Figures 1–3)

*MycoBank*: 847,627

*Type*: South Africa, Knysna – Amatole Afrotemperate region. Collected from a fallen decomposing *Rapanea melanophloeos* log, 3 January 2021, B. Van der Merwe. (Holotype: Dried specimen) deposited for long-term storage at the PREM herbarium, PREM 63384. GenBank accession numbers OP458325 (ITS) and OP458328 (28S).

*Etymology*: Named after the poem Ophélie, by Arthur Rimbaud. Lines such as “long veils … a white phantom … beautiful as snow” seem to be an apt description for the cascading fruiting bodies.

*Description: Basidiocarp* large (300 mm x 60 mm). Singular – growing from the side of a decaying, standing log. Coral like, soft, and fleshy. Cascading clusters of spines stemming from elongated branches. Spines varying in length (1 mm – 10 mm). Young spines and fruiting body stark white with a pinkish blush, yellowing in age. *Basidiospores* Length: (2.5 μm – 3.0 μm – 3.5 μm) Width: (2.0 μm – 2.5 μm – 3.0 μm). Smooth, roughly ovaloid in shape. *Basidia* Length: (15.5 μm – 16.5 μm) Width: (5.0 μm – 7.0 μm). Clavate. *Gloeocystidia* width (7.5 μm – 9.5 μm). Elongated, irregularly textured.

## Discussion

The species of *Hericium* found in the Knysna – Amatole forests was long thought to be *H. coralloides* (Inaturalist, 2022). However, observed differences in morphological and environmental characteristics warranted further study. Fruiting bodies were sequenced in early 2022, and phylogenetic and morphological analysis indicated that *H. ophelieae* sp. nov. is a previously undescribed species. This is unsurprising, as the genus *Hericium* contains cryptic species complexes, with species that are often only accurately identified through phylogenetic studies (Hallenberg, Nilsson and Robledo, 2013).

Locally, *H. ophelieae* sp. nov. has been recorded on hardwoods indigenous to the Knysna-Amatole Southern Afrotemperate region of South Africa. Host trees include, but are not necessarily limited to, *Rapanea melanophloeos* (Cape Beech), *Ilex mitis* (Cape Holly), *Olea capensis* (Ironwood), and *Ocotea bullata* (Stinkwood) trees. *Olea capensis* is endemic to the Southern Afrotemperate forest region, and the other three host trees (*Rapanea melanophloeos, Ilex mitis*, and *Ocotea bullata*) are indigenous to Southern Africa (Van Wyk and Van Wyk 1997). *H. ophelieae* sp. nov. forms fruiting bodies from December to March, coinciding with the rainy season of this region. The formation of fruiting bodies seems to be coupled to heavy rains, humidity, and warmer temperatures (Herrmann, Pers. Comm.).

*H. ophelieae* sp. nov. shares very similar macro-characteristics with its closest relative, *H. bembedjaense* and other members of the genus *Hericium*. However, apart from the differences in phylogeny, *H. ophelieae* sp. nov. has noticeably larger spores than *H. bembedjaense* (3.0 μm – 2.5 μm vs 2.8 μm – 2.0 μm), as well as wider gloeocystidia (7.5 μm/9.5 μm vs 3.4 μm /6.7 μm). It also appears that *H. ophelieae* sp. nov. differs from other *Hericium* species in substrate choice, as they are growing on hardwoods indigenous to Southern Africa. This is compared to *H. bembedjaense* that grows on *Gilbertiodendron dewevrei* and *H. coralloides* that prefers Fagaceae species (Jumbam et al. 2019). To date, no other *Hericium* species have been found in this region or in South Africa (Kinge et al. 2020).

*H. ophelieae* sp. nov. is the first *Hericium* to be described from Southern Africa, however it is likely not the only species to be found here. Many of the biomes in South Africa are known hotspots for plant diversity, and high plant diversity has been shown to increase fungal diversity (Gao et al. 2013; Shen et al. 2021; Wang et al. 2022). Many novel species could remain undescribed due to the slow development of mycology in South Africa, and Africa as a whole (Crous et al. 2006). As a result, amateur mycologists are often involved in the discovery of new species. Accumulated citizen science projects could provide value to mycology in Africa, like it has in other continents (e.g. Heilmann-Clausen et al. 2019).

This discovery will hopefully prompt more studies in the region and will further our knowledge on local biodiversity and microbial – environmental interactions. Reports like these can also have far reaching impacts as *Hericium* members are well known for their production of secondary metabolites that are at the centre of a fast-growing drug discovery field. Further studies into the medicinal properties of *Hericium ophelieae* sp. nov. will hopefully elucidate the potential applications of this novel, medically relevant fungus.
